# Simultaneous confidence intervals that are compatible with closed testing in adaptive designs

**DOI:** 10.1093/biomet/ast035

**Published:** 2013-12-01

**Authors:** D. MAGIRR, T. JAKI, M. POSCH, F. KLINGLMUELLER

**Affiliations:** Department of Mathematics and Statistics, Lancaster University, Lancaster LA1 4YF, U.K.; Center for Medical Statistics, Informatics and Intelligent Systems, Medical University of Vienna, 1090 Wien, Austria

**Keywords:** Closed testing principle, Combination test, Conditional error, Multiple comparisons, Simultaneous inference

## Abstract

We describe a general method for finding a confidence region for a parameter vector that is compatible with the decisions of a two-stage closed test procedure in an adaptive experiment. The closed test procedure is characterized by the fact that rejection or nonrejection of a null hypothesis may depend on the decisions for other hypotheses and the compatible confidence region will, in general, have a complex, nonrectangular shape. We find the smallest cross-product of simultaneous confidence intervals containing the region and provide computational shortcuts for calculating the lower bounds on parameters corresponding to the rejected null hypotheses. We illustrate the method with an adaptive phase II/III clinical trial.

## 1. Introduction

For experiments designed to make inference about a parameter vector *θ* = (*θ*_1_, … , *θ_K_*), it is common to find confidence intervals for all of the individual *θ_k_* such that the simultaneous coverage probability is at least 1 − *α*. Sometimes, though, an experimenter will only attempt to assert that an individual parameter exceeds a specific value, say *θ_k_* > *δ_k_*. If this cannot be achieved in such a way that the probability of making at least one incorrect rejection in a family of hypotheses *H_k_* = {*θ_k_* ⩽ *δ_k_*} (*k* = 1, … , *K*) is no greater than *α*, the experimenter will not assert anything about *θ_k_*. The latter method of inference is used in so-called closed test procedures (Marcus et al., 1976), and its advantage is often greater power.

For experiments conducted in a single stage, Hayter & Hsu (1994) showed how simultaneous 100(1 − *α*)% confidence intervals can be constructed to be compatible with some commonly used closed test procedures, in the sense that a null hypothesis *H_k_* is rejected at familywise level *α* if and only if the confidence interval for *θ_k_* excludes all values for which *H_k_* is true. Often, these intervals are scarcely more informative than the test decisions. For example, for one-sided problems where larger parameter values are more beneficial, no 100(1 − *α*)% lower confidence bound for any individual *θ_k_* can exceed *δ_k_* unless all hypotheses *H*_1_, … , *H_K_* can be rejected at familywise level *α*.

In this article we derive confidence intervals for adaptive experiments. Our motivating example is a seamless phase II/III clinical trial, although the method is not limited to this setting. Such trials consist of a first stage in which *K* experimental treatments, indexed by *T*_1_= {1, … , *K*}, are compared with a common control and, after an interim analysis, a second stage in which only a subset of treatments, indexed by *T*_2_ ⊆ *T*_1_, are compared with the control. The state-of-the-art methodology for this problem (Bauer & Kieser, 1999; Posch et al., 2005; Bretz et al., 2009) is a hybrid of the closure principle of Marcus et al. (1976) and a *p*-value combination which goes back to Fisher (1932). This methodology allows any subset of treatments to be chosen at interim, based on all trial data and external factors. Other adaptations, such as sample size re-estimation, are also possible. A serious concern, though, is that there is no established method for constructing confidence intervals. As emphasized in the International Conference on Harmonisation’s E9 guideline (ICH E9 Expert Working Group, 1999, p. 1932), ‘Estimates of treatment effect should be accompanied by confidence intervals, whenever possible, and the way in which these will be calculated should be identified.’

Posch et al. (2005) proposed 100(1 − *α*)% simultaneous confidence intervals following such a trial. Unfortunately, their intervals are not guaranteed to be compatible with the closed test procedure. Here, we construct intervals that are compatible. As in the one-stage case, an inevitable shortcoming of these intervals is that they are not always substantially more informative than the original test decisions. We will show that this problem is mitigated to some extent by the adaptive nature of the experiment.

## 2. Fundamental Methodology

### 2·1. Closure principle

The closure principle of Marcus et al. (1976) is a general method for multiple hypothesis testing. A formal description is given in Finner & Strassburger (2002), and we adopt similar notation here. Let $P=\{P_{\theta^{*}}:\theta^{*}\in \Theta \}$ be a family of probability measures defined on a common sample space (Ω, F), where Θ is a multi-dimensional parameter space. Suppose that we wish to test a family of null hypotheses $H=\{H_{i}:i\in I\}$, where *H_i_* ⊂ Θ for each *i* in some index set I. Let $\psi =\{\psi_{i}:i\in I\}$ denote a multiple test of H, with each component *ψ_i_* taking value 0 or 1 corresponding to nonrejection or rejection of *H_i_*, respectively. It is often desirable to ensure that

(1) $$\underset{\theta^{*}\in \Theta }{\sup}P_{\theta^{*}}\left(\underset{i\in I\left(\theta^{*}\right)}{⋃}\{\psi_{i}=1\}\right)⩽\alpha ,$$

where $I\left(\theta^{*}\right)=\{i\in I:\theta^{*}\in H_{i}\}$ is the index set of true hypotheses under θ*. In other words, the probability of rejecting at least one true null hypothesis is bounded by *α*. This is known as strong control of the familywise error rate. The closure principle can be used to ensure (1). We are required to find, for each I⊆I such that $H_{I}={⋂}_{i\in I}H_{i}$ is nonempty, a local level-*α* test *φ_I_* for the intersection hypothesis *H_I_*; that is, we require

(2) $$\underset{\theta^{*}\in H_{I}}{\sup}P_{\theta^{*}}\left(\varphi_{I}=1\right)⩽\alpha ,$$

where *φ_I_* takes values in {0, 1} with the usual interpretation. If we define $\psi_{i}=\min_{I:H_{I}\neq \emptyset ,H_{I}\subseteq H_{i}}\left(\varphi_{I}\right)$, then (1) holds. This can be very useful, as in many applications it is easy to find tests satisfying (2), whereas validating (1) directly is hard.

### 2·2. Combination test

Fisher (1932) discussed combining independent *p*-values to test a single null hypothesis. For convenience and brevity, we will only consider two-stage designs. We define a *p*-value combination function *Q*: [0, 1]^2^ ↣ [0, 1] that is left-continuous and nondecreasing in both its arguments and is uniformly distributed provided that both arguments are themselves independent and uniformly distributed. An example is

(3) $$Q\left(u,v\right)=1-\Phi \left[2^{1∕2}\left\{\Phi^{-1}\left(1-u\right)+\Phi^{-1}\left(1-v\right)\right\}\right],$$

where Φ denotes the standard normal distribution function.

Such a combination function lends itself to a two-stage adaptive closed test, *ψ*, for a family of null hypotheses, H. An important application, discussed in Bretz et al. (2009), is a seamless phase II/III confirmatory clinical trial. We henceforth restrict attention to a parameter *θ* = (*θ*_1_, … , *θ_K_*) taking values in parameter space $\Theta ={\mathbb{R}}^{K}$ and a family of null hypotheses $H=\{H_{k}:k\in T_{1}\}$ where *T*_1_ = {1, … , *K*} and *H_k_* = {*θ_k_* ⩽ *δ_k_*} (*k* ∈ *T*_1_) for some constants $\delta_{1},\ldots ,\delta_{K}\in \mathbb{R}$. The *θ_k_* (*k* ∈ *T*_1_) might correspond to the mean effects of *K* different treatments, for example. By defining local tests *φ_I_* (*I* ⊆ *T*_1_) via a combination function Q, it is possible to make data-dependent modifications to the trial design at an interim analysis (cf. Bauer & Kieser, 1999; Hommel, 2001; Brannath et al., 2002). For instance, attention can be focused on a subset *T*_2_ ⊆ *T*_1_ of the initial hypotheses of interest; changes can be made to sample sizes, allocation ratios, etc.

### 2·3. Two-stage closed test procedure

Assume that the full first-stage trial data are represented by a random vector $X\in {\mathbb{R}}^{n}$ with distribution function *G*(*x*; *θ*). Prior to starting the trial, one must specify a combination function Q and, for each *I* ⊆ *T*_1_, a first-stage test of $H_{I}={⋂}_{i\in I}$ *H_i_* with an associated *p*-value function $p_{I}^{\left(1\right)}:{\mathbb{R}}^{n}\to [0,1]$ that satisfies $\sup_{\theta^{*}\in H_{I}}\int_{\left\{p_{I}^{\left(1\right)}\left(x\right)⩽u\right\}}dG\left(x;\theta^{*}\right)⩽u$ for all u∈[0,1]. The second-stage design is unspecified.

At the interim analysis, the experimenter defines a second-stage design, *d*, by choosing a subset of the original hypotheses, indexed by *T*_2_ ⊆ *T*_1_, to continue studying in the second stage, along with second-stage sample sizes and, for each *I* ⊆ *T*_1_, a second-stage hypothesis test for *H_I_*. See below for a proposal for choosing second-stage tests for *H_I_* where *I* ⊈ *T*_2_. We assume that the design *d* is allowed to depend on the unblinded first-stage data *x* without prespecifying an adaptation rule. Let *Y* denote the data collected at the second stage, taking values in ${\mathbb{R}}^{m}$, and let $p_{I,x,d}^{\left(2\right)}\left(y\right)$ (*I* ⊆ *T*_1_) denote the *p*-value functions of the second-stage tests. Because the tests used in the second stage depend on the first-stage data *x* and the chosen design *d*, the *p*-value functions will in general depend on both.

Let *F_x,d_*(*y*; *θ*) denote the distribution function of the second-stage data, given the chosen design *d* and interim data *x*. We assume that for all *x, d* and *I* ⊆ *T*_1_, the second-stage *p*-values $p_{I,x,d}^{\left(2\right)}$ satisfy $\sup_{\theta^{*}\in H_{I}}\int_{\left\{p_{I,x,d}^{\left(2\right)}\left(y\right)⩽u\right\}}dF_{x,d}\left(y;\theta^{*}\right)⩽u$ for all *u* ∈ [0, 1]. The distribution *F_x,d_* is assumed to be known, i.e., not merely specified up to a null set, for all *x* and *d*, a condition that can be formalized by assuming an appropriate regression model (Brannath et al., 2012). See § 3·2 for a numerical example.

At the final analysis, for each *I* ⊆ *T*_1_, the test decision is *φ_I_* = 1 if and only if $Q\{p_{I}^{\left(1\right)},p_{I,x,d}^{\left(2\right)}\}⩽\alpha$. As shown in Brannath et al. (2012), this combination test for *H_I_* controls the Type I error rate at level *α*.

We assume that only data for the hypotheses indexed by *T*_2_ are collected in the second stage and propose setting $p_{I}^{\left(2\right)}=p_{I\cap T_{2}}^{\left(2\right)}$ for *I* ⊈ *T*_2_, where we drop the indices *x* and *d* for simplicity and set $p_{\emptyset }^{\left(2\right)}=1$ by convention. Such second-stage *p*-values have the required distribution under *H*_*I*∩*T*_2__ and hence also under *H_I_*.

We emphasize that while Type I error control is guaranteed even if the second-stage design is initially open-ended, in the design of actual clinical trials it is crucial to perform detailed planning based on likely first-stage outcomes. The added flexibility is necessary because it is impossible to foresee all eventualities in extremely complex areas such as clinical drug development.

## 3. Confidence regions

### 3·1. Partitioning the parameter space

A standard approach to deriving a 100(1 − *α*)% confidence set for *θ* is to perform a level-*α* test of each elementary hypothesis {*θ* = θ*} (θ* ∈ Θ) and include all θ* corresponding to nonrejected hypotheses (see, e.g., Lehmann, 1986, p. 90). To ensure compatibility with closed testing, the key idea (Stefansson et al., 1988; Hayter & Hsu, 1994; Finner & Strassburger, 2002) is to partition the parameter space into disjoint regions

$$\Theta_{I}=\{\theta^{*}\in \Theta :\theta_{i}^{*}⩽\delta_{i},i\in I;\theta_{i}^{*}>\delta_{i},i\in T_{1}\backslash I\}\;\left(I\subseteq T_{1}\right)$$

and apply different tests in each of the disjoint Θ_*I*_. If, for each *I* ⊆ *T*_1_, we let {*φ_I_* (θ*): θ* ∈ Θ} denote a family of tests with

(4) $$\underset{\theta^{*}\in \Theta }{\inf}P_{\theta^{*}}\{\varphi_{I}\left(\theta^{*}\right)=0\}⩾1-\alpha ,$$

where *φ_I_* (θ*) takes values in {0, 1} with the usual interpretation, we can apply the following general result from Hsu (1996, p. 234).

Lemma 1. *A level*-100(1 − *α*)% *confidence set for θ is*

(5) $$C=\underset{I\subseteq T_{1}}{⋃}\left[\{\theta^{*}\in \Theta :\varphi_{I}\left(\theta^{*}\right)=0\}\cap \Theta_{I}\right].$$

Our aim is to find families of tests such that *C* is compatible with the two-stage closed test procedure. This requires us to augment our specification of $p_{I\cap T_{j}}^{\left(j\right)}\left(j=1,2;I\subseteq T_{1}\right)$ with a family of *p*-values $\left\{p_{I\cap T_{j}}^{\left(j\right)}\left(\theta^{*}\right):\theta^{*}\in \Theta \right\}$ where, under {*θ* = θ*}, the distribution of $p_{I}^{\left(1\right)}\left(\theta^{*}\right)$ and $p_{I\cap T_{2}}^{\left(2\right)}\left(\theta^{*}\right)$ meet conditions as outlined for $p_{I}^{\left(1\right)}$ and $p_{I\cap T_{2}}^{\left(2\right)}$ in § 2·3. Additionally, if we treat the data as fixed and view each family as a function $p_{I\cap T_{j}}^{\left(j\right)}:\Theta \to [0,1]$, then unless $I\cap T_{j}=\emptyset ,p_{I\cap T_{j}}^{\left(j\right)}\left(\theta^{*}\right)$ is constant in all arguments $\theta_{i}^{*}$ such that *i* ∉ *I* ∩ *T_j_*, and is left-continuous and nondecreasing in all arguments $\theta_{i}^{*}$ such that *i* ∈ *I* ∩ *T_j_*, with $p_{I\cap T_{j}}^{\left(j\right)}\left(\theta^{*}\right)=p_{I\cap T_{j}}^{\left(j\right)}$ for any θ* such that $\theta_{i}^{*}=\delta_{i}$ for all *i* ∈ *I* ∩ *T_j_*. Furthermore, we assume that

(6) $$\lim_{\theta_{i}^{*}\to \infty ,i\in T_{2}}p_{\emptyset }^{\left(2\right)}\left(\theta^{*}\right)=1.$$

Proposition 1. *Inserted into* (5), *the following families of hypothesis tests give rise to a* 100(1 − *α*)% *confidence set for θ, denoted by C, that is compatible with the two-stage closed test procedure, i.e., ψ_k_* = 1 *if and only if H_k_* ∩ *C* = ∅: *for* ∅ ≠ *I* ⊆ T_1_ and θ* ∈ Θ,

(7) φI(θ∗)={1,Q{pI(1)(θ∗),pI∩T2(2)(θ∗)}⩽α,0,Q{pI(1)(θ∗),pI∩T2(2)(θ∗)}>α,}

*and* {*φ_∅_*(θ*): θ* ∈ Θ *} is any family of tests satisfying* (4).

*Proof.* See the Appendix.

There will be no unique collection of families of *p*-values satisfying the aforementioned distributional and monotonicity constraints. Rather, the families must be specified in a two-stage procedure in an analogous way to the *p*-values in § 2·3. As will become clear from the example below, for many commonly encountered scenarios and when *I* ∩ *T_j_* ≠ ∅, the choice of $\left\{p_{I\cap T_{j}}^{\left(j\right)}\left(\theta^{*}\right):\theta^{*}\in \Theta \right\}$ will be obvious from the choice of $p_{I\cap T_{j}}^{\left(j\right)}$. As a simple example, suppose that $p_{\left\{k\right\}}^{\left(j\right)}$ is the *p*-value from a one-sided *z*-test of the null hypothesis {*θ_k_* ⩽ *δ_k_*} using the stage-*j* data only. Then the natural choice for $p_{\left\{k\right\}}^{\left(j\right)}\left(\theta^{*}\right)$ is the one-sided *p*-value from a standard *z*-test of $\left\{\theta_{k}⩽\theta_{k}^{*}\right\}$ using the same stage-*j* data.

While for *I* ∩ *T_j_* ≠ ∅ there will often be a natural choice for $p_{I\cap T_{j}}^{\left(j\right)}\left(\theta^{*}\right)$, it is unclear how *φ_∅_*(θ*) and $p_{\emptyset }^{\left(2\right)}\left(\theta^{*}\right)$ should be chosen. A reasonable suggestion is given below.

Corollary 1. *Define* $p_{\emptyset }^{\left(j\right)}\left(\theta^{*}\right)=p_{T_{j}}^{\left(j\right)}\left(\theta^{*}\right)$ *for j* = 1,2. *The following is a* 100(1 − *α*)% *confidence region for θ that is compatible with the two-stage closed test procedure:*

(8) $$C_{1}=\underset{I\subseteq T_{1}}{⋃}\left[\theta^{*}\in \Theta_{I}:Q\{p_{I}^{\left(1\right)}\left(\theta^{*}\right),p_{I\cap T_{2}}^{\left(2\right)}\left(\theta^{*}\right)\}>\alpha \right].$$

The properties of a region defined by (8) are best illustrated by a specific example.

### 3·2. Example

Posch et al. (2005) considered a clinical trial where three active treatments, indexed by *T*_1_ = {*A, B, C*}, are compared with a placebo using a two-stage adaptive design. The individual null hypotheses of interest are *H_k_* = {*θ_k_* ⩽ 0} (*k* ∈ *T*_1_), where *θ_k_* = *π_k_* − *π*_0_ denotes the difference between the success probabilities of treatment *k* and placebo. Denote the observed success rate of treatment *k* in stage *j* by ${\hat{\pi }}_{k,j}\left(k\in T_{1}\cup \{0\};\;j=1,2\right)$, where treatment 0 corresponds to a placebo.

At the design stage, the inverse normal combination function (3) is specified and *n*_1_ = 140 first-stage patients are recruited to each treatment arm. Approximately, the ${\hat{\theta }}_{k,1}={\hat{\pi }}_{k,1}-{\hat{\pi }}_{0,1}\left(k\in T_{1}\right)$ are multivariate normal with $E\left({\hat{\theta }}_{k,1}\right)=\theta_{k},\;\mathrm{var}\left({\hat{\theta }}_{k,1}\right)=\{{\hat{\pi }}_{k,1}\left(1-{\hat{\pi }}_{k,1}\right)+{\hat{\pi }}_{0,1}\left(1-{\hat{\pi }}_{0,1}\right)\}∕n_{1}$ and positive correlations. Based on this assumption, Simes (1986) tests are used for each intersection hypothesis; that is, $p_{\left\{k\right\}}^{\left(1\right)}=1-\Phi [{\hat{\theta }}_{k,1}{\left\{\mathrm{var}\left({\hat{\theta }}_{k,1}\right)\right\}}^{-1∕2}]$ for *k* ∈ *T*_1_ and, for |*I*|>1, $p_{I}^{\left(1\right)}=\min_{k\in I}\;p_{\left\{k\right\}}^{\left(1\right)}|I|∕R\left(k,I\right)$, where *R*(*k*, *I*) denotes the rank of $p_{\left\{k\right\}}^{\left(1\right)}$ among $\left\{p_{\left\{i\right\}}^{\left(1\right)}:i\in I\right\}$. The natural way of augmenting these *p*-values is to define $p_{\left\{k\right\}}^{\left(1\right)}\left(\theta^{*}\right)=1-\Phi [\left({\hat{\theta }}_{k,1}-\theta_{k}^{*}\right){\left\{\mathrm{var}\left({\hat{\theta }}_{k,1}\right)\right\}}^{-1∕2}]$ for *k* ∈ *T*_1_ and $p_{I}^{\left(1\right)}\left(\theta^{*}\right)=\min_{k\in I}\;p_{\left\{k\right\}}^{\left(1\right)}\left(\theta^{*}\right)|I|∕R\left(k,I,\theta^{*}\right)$ for |*I*| > 1, where *R*(*k*, *I*, θ*) denotes the rank of $p_{\left\{k\right\}}^{\left(1\right)}\left(\theta^{*}\right)$ among $\left\{p_{\left\{i\right\}}^{\left(1\right)}\left(\theta^{*}\right):i\in I\right\}$.

Suppose that the unblinded first-stage results are ${\hat{\pi }}_{0,1}=0\cdot 21,{\hat{\pi }}_{A,1}=0\cdot 22$, ${\hat{\pi }}_{B,1}=0\cdot 3$ and ${\hat{\pi }}_{C,1}=0\cdot 36$. The experimenter decides that treatments *A* and *C* are not to be considered in the second stage owing to lack of efficacy and safety concerns, respectively. A further *n*_2_ = 140 patients are recruited to both treatment *B* and placebo. A family of *p*-values with $p_{\left\{B\right\}}^{\left(2\right)}\left(\theta^{*}\right)=1-\Phi [({\hat{\theta }}_{B,2}-\theta_{B}^{*}){\left\{\mathrm{var}({\hat{\theta }}_{B,2})\right\}}^{-1∕2}]$ is chosen, where ${\hat{\theta }}_{B,2}={\hat{\pi }}_{B,2}-{\hat{\pi }}_{0,2}$.

Now suppose that the second-stage results are ${\hat{\pi }}_{0,2}=0\cdot 19$ and ${\hat{\pi }}_{B,2}=0\cdot 31$. The *p*-values from the elementary hypotheses are $p_{\left\{A\right\}}^{\left(1\right)}=0\cdot 419$, $p_{\left\{B\right\}}^{\left(1\right)}=0\cdot 0412$, $p_{\left\{C\right\}}^{\left(1\right)}=0\cdot 00241$ and $p_{\left\{B\right\}}^{\left(2\right)}=0\cdot 00961$. Therefore $p_{\left\{A,B,C\right\}}^{\left(1\right)}=3p_{\left\{C\right\}}^{\left(1\right)}$, $p_{\left\{A,B\right\}}^{\left(1\right)}=2p_{\left\{B\right\}}^{\left(1\right)}$ and $p_{\left\{B,C\right\}}^{\left(1\right)}=2p_{\left\{C\right\}}^{\left(1\right)}$. As $\min_{I\subseteq }T_{1},B\in IQ(p_{I}^{\left(1\right)},p_{B}^{\left(2\right)})\leq 0\cdot 025$, *H_B_* can be rejected at familywise level 0·025. Both *H_A_* and *H_C_* fail to be rejected, as $Q\{p_{\left\{k\right\}}^{\left(1\right)},1\}=1$ for *k* = *A*,*C*. A compatible 97·5% confidence region for *θ* is given by

(9) $$\underset{I\subseteq T_{1}}{⋃}\left\{\theta^{*}\in \Theta_{I}:Q\{p_{I}^{\left(1\right)}\left(\theta^{*}\right),p_{B}^{\left(2\right)}\left(\theta^{*}\right)\}>0.025\right\},$$

where $p_{\emptyset }^{\left(1\right)}\left(\theta^{*}\right)$ is defined as $p_{T_{1}}^{\left(1\right)}\left(\theta^{*}\right)$ for all θ* ∈ Θ.

**Fig. 1:**
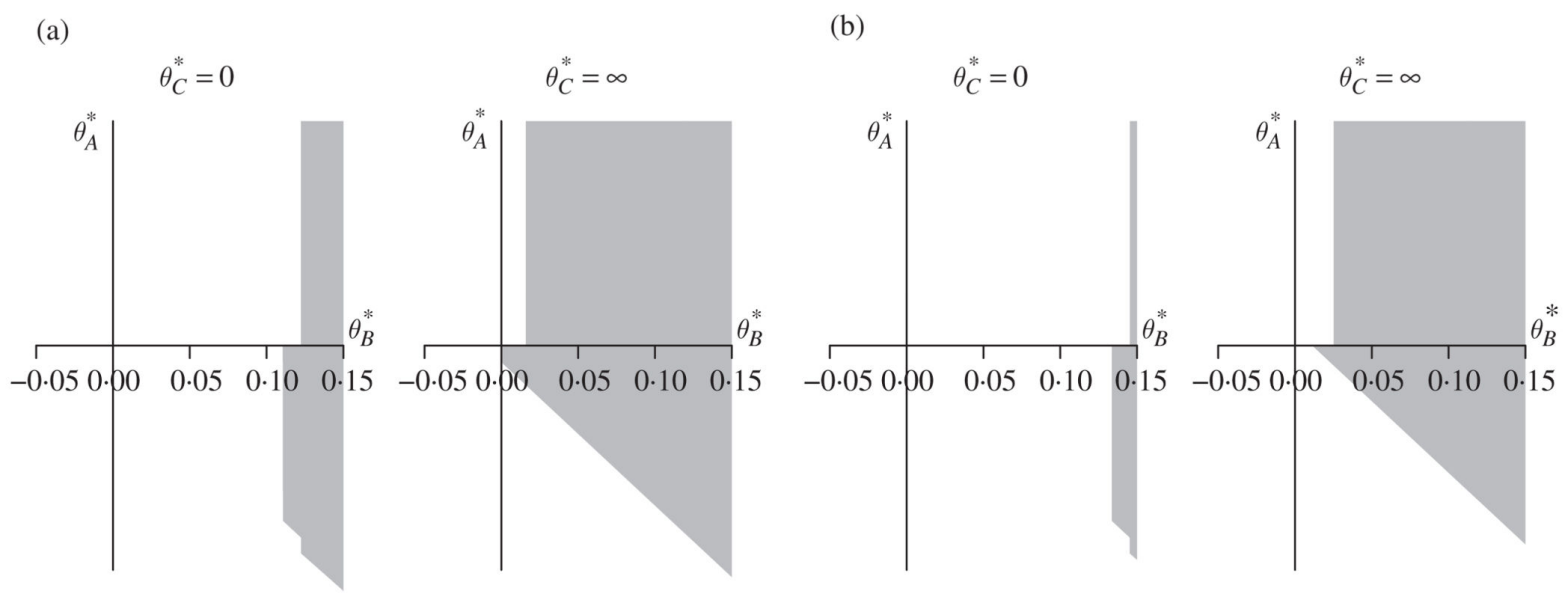
Cross-sections of confidence regions of the form (9) for making inference on the second-stage parameter of interest, *θ_B_*, in the example of § 3·2: (a) two cross-sections of the 97·5% confidence region; (b) two cross-sections of the 95% confidence region.

The region (9) will have a complicated three-dimensional shape. However, in terms of making inference on *θ_B_*, its crucial features can be seen by taking two cross-sections, as displayed in Fig. 1. As $p_{I}^{\left(1\right)}\left(\theta^{*}\right)$ is nondecreasing in $\theta_{C}^{*}$ for all *I* ⊆ *T*_1_, we know that for any *γ* ∈ (-∞, 0), the cross-section at $\theta_{C}^{*}=\gamma$ is contained in the cross-section at $\theta_{C}^{*}=0$. Similarly, for any *γ* ∈ (0, ∞), the cross-section at $\theta_{C}^{*}=\gamma$ is contained in the limit of the cross-section of the region as $\theta_{C}^{*}\to \infty$. One can see immediately from Fig. 1 that for any *ϵ* > 0, the 97·5% confidence region fails to exclude all parameter vectors θ* such that $\theta_{B}^{*}⩽\epsilon$. In other words, the lower confidence bound on *θ_B_* provides no more information than the decision of the closed test procedure.

For confidence intervals that are compatible with single-stage closed test procedures (Hayter & Hsu, 1994; Strassburger & Bretz, 2008; Guilbaud, 2008), a necessary condition for obtaining informative lower confidence bounds for parameters corresponding to the rejected null hypotheses is that *ψ_k_* =1 for all *k* ∈ *T*_1_. In the adaptive setting, this is no longer a necessary condition. For example, repeating the above test procedure at level *α*=0·05, the compatible 95% confidence region analogous to (9) is also summarized in Fig. 1. Here it appears, and indeed can be verified by considering all values of $\theta_{A}^{*}$, that there does exist some *ϵ* > 0 such that the confidence region excludes all parameter vectors θ* for which $\theta_{B}^{*}⩽\epsilon$. We will show that for the two-stage adaptive setting, a necessary condition for informative lower confidence bounds on parameters corresponding to the rejected null hypotheses is that *ψ_k_* =1 for all *k* ∈ *T*_2_. However, as can be seen from Fig. 1, this condition is not sufficient.

### 3·3. A two-stage, single-step confidence region

Posch et al. (2005) proposed the following 100(1 − *α*)% confidence region:

(10) $$C_{2}=\left\{\theta^{*}\in \Theta :Q\{p_{T_{1}}^{\left(1\right)}\left(\theta^{*}\right),p_{T_{2}}^{\left(2\right)}\left(\theta^{*}\right)\}>\alpha \right\}.$$

They note that the resulting confidence intervals are not compatible with the closed test procedure described in § 2·3 (Posch et al., 2005, p. 3702). Nevertheless, the region (10) can be used to generate an alternative multiple test. More generally, any 1 − *α* confidence set *C* generates a multiple test for a family of hypotheses H, whereby $H_{k}\in H$ is rejected if and only if *H_k_* ∩ *C* = ∅. This guarantees strong control of the familywise error rate (1). The multiple test generated by (10) can be thought of as single-step in the sense that rejection or nonrejection of a null hypothesis does not take into account the decision for any other hypothesis. If *H_k_* is rejected, informative lower bounds will be available for *θ_k_* regardless of the test decisions for all other hypotheses.

## 4. Computation of confidence intervals

### 4·1. Least-favourable parameter configurations

In the above example, marginal inference on *θ_B_* was achieved by considering least-favourable parameter configurations for *θ_k_*, *k* ∈ *T*_1_ \ {*B*}. This idea can be generalized to find 100(1 − *α*)% simultaneous confidence intervals containing (8) or (10).

Definition 1. *For j* = 1, 2, *k* ∈ *T*_1_ *and* *I* ⊆ *T_j_*, *the locally least-favourable jth-stage p-value function for H_k_ in* Θ*_I_*, $p_{k,I}^{\left(j\right)}:\mathbb{R}\to [0,1]$, *is defined for I* ≠ ∅ *as* $p_{k,I}^{\left(j\right)}\left(\vartheta \right)=p_{I}^{\left(j\right)}\left(\xi \right)$, *where ξ* =(*ξ*_1_, … , *ξ_K_*) *with ξ_i_* =δ_i_ for i ≠ k and *ξ*_k_ = *ϑ*. *Additionally, for j* = 1, 2,

(11) $$p_{k,\emptyset }^{\left(j\right)}\left(\vartheta \right)=\lim_{\xi_{i}\to \infty ,i\in T_{j}\backslash \{k\}}p_{T_{j}}^{\left(j\right)}\left(\xi \right)\;\left(\xi_{k}=\vartheta \right).$$

Proposition 2. *The smallest Cartesian product of intervals*, ×_*k*∈*T*_1__(*l_k_*, ∞), *that contains the confidence region* (8) *has l_k_* = min_*I*⊆*T*_1__ *l_k,I_*, *where for k* ∈ *I*,

(12) lk,I={∞(φI=1),sup{ϑ:Q{pk,I(1)(ϑ),pk,I∩T2(2)(ϑ)}⩽α}(φI=0),}

*and for k* ∉ *I*,

(13) $$l_{k,I}=\max\left(\delta_{k},\text{sup}\left\{\vartheta :Q\{p_{k,I}^{\left(1\right)}\left(\vartheta \right),p_{k,I\cap T_{2}}^{\left(2\right)}\left(\vartheta \right)\}⩽\alpha \right\}\right).$$

*Furthermore, these intervals are compatible with the two-stage closed test procedure, i.e., ψ_k_* = 1 *if and only if H_k_* ∩ ×_*k*∈*T*_1__(*l_k_*, ∞)=∅.

*Proof*. See the Appendix.

In general, to find each interval requires one-dimensional root finding for each *I* ⊆ *T*_1_, a calculation that is *O*(2^*K*^). However, substantial shortcuts are available for reducing the computational burden.

### 4·2. Efficient computation of confidence bounds

There are two possible scenarios at the end of the closed test procedure: either *ψ_k_* = 1 for all *k* ∈ *T*_2_, or at least one *H_k_* (*k* ∈ *T*_2_) fails to be rejected. In the latter case, there exists some *I* ⊆ *T*_1_ with *I* ∩ *T*_2_ ≠ ∅ such that for any *k* ∈ *T*_2_,

$$\alpha <Q\left(p_{I}^{\left(1\right)},p_{I\cap T_{2}}^{\left(2\right)}\right)=Q\left\{p_{k,I}^{\left(1\right)}\left(\delta_{k}\right),p_{k,I\cap T_{2}}^{\left(2\right)}\left(\delta_{k}\right)\right\}$$

and therefore *l_k_* ⩽ *l_k,I_* ⩽ *δ_k_*. Due to the compatibility of the intervals with the closed test procedure, if *ψ_k_* = 1, then *l_k_* = *δ_k_*; if *ψ_k_* = 0, then *l_k_* < *δ_k_*.

If *ψ_k_* =1 for all *k* ∈ *T*_2_, then *l*_k_ ⩾ *δ_k_* for all *k* ∈ *T*_2_. Additionally, we can use the fact that for all *k* ∈ *T*_2_ and *I* ⊆ *T*_1_ with *I* ∩ *T*_2_ ≠ ∅, we know from (12) and (13) that *l_k,I_* = ∞; so, when finding *l_k_* =min_*I*⊆*T*_1__ *l_k,I_* in Proposition 2, the minimum can be taken over a much smaller number of *l_k,I_*. The following algorithm finds the lower bounds for all parameters corresponding to the rejected hypotheses.

*Step* 1. Perform the closed test procedure. If *ψ_k_′* = 0 for some *k*′ ∈ *T*_2_, then *l_k_* = *δk* for *ψ_k_* =1 and *l_k_* < *δ_k_* for *ψ_k_* =0. If *ψ_k_* =1 for all *k* ∈ *T*_2_, go to Step 2.

*Step* 2. Find $p_{M}=\max_{\emptyset \neq I\subseteq T_{1}\backslash T_{2}}p_{I}^{\left(1\right)}$. If *T*_1_ \ *T*_2_ = ∅, then *p*_M_ =0.

*Step* 3. For *k* ∈ *T*_2_,

$$l_{k}=\max\left[\delta_{k},\text{sup}\left\{\vartheta :Q\left[\max\{p_{M},p_{k,\emptyset }^{\left(1\right)}\left(\vartheta \right)\},p_{k,\emptyset }^{\left(2\right)}\left(\vartheta \right)\right]⩽\alpha \right\}\right].$$

The cost of computing the intervals for *θ_k_* (*k* ∈ *T*_2_) in Step 3 is linear in the number of parameters. Step 1 is *O*(2^|*T*_1_|^), but a shortcut of *O*(|*T*_1_|^2^) is given in Brannath & Bretz (2010). Step 2 is *O*(2^|T_1_\*T*_2_|^), but a shortcut of size |*T*_1_ \ *T*_2_| is available, provided there exists an ordering *i*_1_, … , *i_k_* of *T*_1_ \ *T*_2_ such that for each *u* ∈ {1, … , *k*}, $p_{J}^{\left(1\right)}⩽p_{L}^{\left(1\right)}$ for all *J* ⊆ *L* ⊆ {*i_u_*, … , *i_k_*} with *i_u_* ∈ *J*. This is because we only have to check $p_{\left\{i_{u},\ldots ,i_{k}\right\}}$ for *u* =1, … , *k*. Many common multiple test procedures, such as those based on Dunnett (1955) tests or weighted Bonferroni tests, satisfy this condition, with the ordering *i*_1_, … , *i*_k_ following the ordering of the univariate test statistics or the weighted elementary *p*-values (Brannath & Bretz, 2010).

### 4·3. Lower bounds for parameters corresponding to retained hypotheses

Consider *k* ∈ *T*_2_ such that *ψ_k_* = 0. We know that *l_k_* < *δ_k_*, and therefore we need only consider *l_k,I_* such that *k* ∈ *I*. However, since in general *l_k,I_* < ∞, finding the minimum such lower bound will still have a computational cost that is exponential in the number of parameters.

For *k* ∈ *I* ⊆ *T*_1_ \ *T*_2_, we have $p_{k,I\cap T_{2}}^{\left(2\right)}\left(\vartheta \right)=p_{k,\emptyset }^{\left(2\right)}\left(\vartheta \right)$ and know from (11) and (6) that this is equal to 1. Many commonly used combination functions, including (3), have the property that *v* = 1 implies Q(u,v)=1. In this case, *l_k_* = −∞ for all *k* ∈ *T*_1_ \ *T*_2_.

### 4·4. Lower bounds for the two-stage single-step procedure

Posch et al. (2005) showed that the region (10) is contained in a rectangle, $\times_{k\in T_{1}}({\overset{‒}{l}}_{k},\infty )$, where

(14) $${\overset{‒}{l}}_{k}=\text{sup}\left\{\vartheta :Q\{p_{k,\emptyset }^{\left(1\right)}\left(\vartheta \right),p_{k,\emptyset }^{\left(2\right)}\left(\vartheta \right)\right\}⩽\alpha \}.$$

The computation of each interval requires only a one-dimensional search for a root, and overall computation will be linear in the number of parameters.

### 4·5. Example continued

Recall from § 3·2 that *T*_2_ = {*B*} and *ψ_B_* = 1. Proceeding to Step 2 of the above algorithm, *p*_M_ =0·419. In this case we need just one iteration in Step 3, because

$$Q\left[\max\{0.419,p_{B,\emptyset }^{\left(1\right)}\left(0\right)\},p_{B,\emptyset }^{\left(2\right)}\left(0\right)\right]=0.0360>0.025,$$

and therefore the 97·5% confidence interval for *θ_B_* is (0, ∞), consistent with Fig. 1. This example emphasizes that there is a price to pay for the additional power of the closed test as opposed to the single-step procedure of § 3·3 with, by (14),

$${\overset{‒}{l}}_{B}=\text{sup}\left\{\vartheta :Q\{p_{B,\emptyset }^{\left(1\right)}\left(\vartheta \right),p_{B,\emptyset }^{\left(2\right)}\left(\vartheta \right)\}⩽0.025\right\}=0.0159.$$

While this agrees with the assertion *θ_B_* > 0 in this specific case, it is invalid to claim it as a 97·5% lower confidence bound if the closed test procedure of § 2·3 had been planned. One can see that for any *α* > 0·036, the 100(1 − *α*)% confidence interval for treatment *B* that is compatible with the closed test procedure has a positive lower bound. For example, the 95% lower confidence bound is *l_B_* = 0·0112, consistent with Fig. 1. Again, if the region (10) had been specified pre-trial, the 95% lower confidence bound (14) would have been ${\overset{‒}{l}}_{B}=0\cdot 0252$.

### 5. Confidence bounds for closed tests based on the conditional error rate

Consider again the two-stage closed test procedure of § 2·3. As an alternative to combination tests, Koenig et al. (2008) used the conditional error approach (Proschan & Hunsberger, 1995) to derive local tests *φ_I_* (*I* ⊆ *T*_1_). The only difference is that instead of prespecifying a combination function *Q* and first-stage *p*-value $p_{I}^{\left(1\right)}$, one must prespecify a measurable conditional error function $A_{I}:{\mathbb{R}}^{n}\to [0,1]$ such that

$$\underset{\theta^{*}\in H_{I}}{\text{sup}}\int_{{\mathbb{R}}^{n}}A_{I}\left(x\right)dG\left(x;\theta^{*}\right)⩽\alpha$$

and, at the final analysis, *φ_I_* =1 if and only if $p_{I\cap T_{2}}^{\left(2\right)}⩽A_{I}\left(x\right)$.

To produce a compatible 100(1 − *α*)% confidence region for *θ*, each *A_I_* (*I* ⊆ *T*_1_) must be augmented with a family of conditional error functions {*A_I_*(θ*) : θ* ∈ Θ} such that $\int_{{\mathbb{R}}^{n}}A_{I}\left(\theta^{*}\right)\left(x\right)dG\left(x;\theta^{*}\right)⩽\alpha$ and, for fixed $x\in {\mathbb{R}}^{n}$, *A_I_*(θ*) is constant in all arguments $\theta_{i}^{*}$ with *i* ∉ *I* and is left-continuous and nonincreasing in all arguments $\theta_{i}^{*}$ with *i* ∈ *I*. Furthermore, *A_I_* (θ*)= *A_I_* for all θ* ∈ Θ such that $\theta_{i}^{*}=\delta_{i}$ for *i* ∈ *I*. The second-stage *p*-values $p_{I\cap T_{2}}^{\left(2\right)}\left(I\subseteq T_{1}\right)$ must be augmented with a family $\left\{p_{I\cap T_{2}}^{\left(2\right)}\left(\theta^{*}\right):\theta^{*}\in \Theta \right\}$ as described in § 3·1.

Müller & Schäfer (2004) propose defining *A_I_* = sup_θ*∈*H*_I__ *E*_θ*_(*ϕ_I_* | *X*), where *ϕ_I_* is a pre-planned fixed sample level-*α* test for *H_I_*. In many situations the natural choice for *A_I_*(θ*) will be obvious from *A_I_*. For example, if *ϕ_I_* is the decision function for a Dunnett (1955) test of *H_I_* = ⋂_*k*∈_I__{*θ_k_* ⩽ *δ_k_*}, then it is natural to choose *A_I_* (θ*) = *E*_θ*_(*ϕ*_*I*,θ*_ | *X*) where *ϕ*_I,θ*_ is the decision function for a Dunnett test of ${⋂}_{k\in I}\{\theta_{k}⩽\theta_{k}^{*}\}$ which can be derived via a corresponding translation of the test statistics.

Using the arguments of Propositions 1 and 2, it can be shown that, analogously to (8), a compatible 100(1 − *α*)% confidence region for *θ* is

$$\underset{I\subseteq T_{1}}{⋃}\left\{\theta^{*}\in \Theta_{I}:p_{I\cap T_{2}}^{\left(2\right)}\left(\theta^{*}\right)>A_{I}\left(\theta^{*}\right)\right\},$$

where $p_{\emptyset }^{\left(2\right)}\left(\theta^{*}\right)$ and *A*_∅_(θ*) are set equal to $p_{T_{2}}^{\left(2\right)}\left(\theta^{*}\right)$ and *A*_*T*_1__(θ*) respectively. Also, the largest compatible 100(1 − *α*)% confidence lower bounds are *l_k_* =min_*I*⊆*T*_1__ *l_k,I_*, where for *k* ∈ *I*,

lk,I={∞(φI=1),sup{ϑ:pk,I∩T2(2)(ϑ)⩽Ak,I(ϑ)}(φI=0),}

and for *k* ∉ *I*, $l_{k,I}=\max[\delta_{k},\text{sup}\{\vartheta :p_{k,I\cap T_{2}}^{\left(2\right)}\left(\vartheta \right)⩽A_{k,I}\left(\vartheta \right)\}]$ with *A_k,I_*(ϑ) defined analogously to $p_{k,I}^{\left(1\right)}\left(\vartheta \right)\left(k\in T_{1};I\subseteq T_{1}\right)$ in Definition 1.

## 6. Concluding remarks

The lower confidence bounds (12)–(13) provide more information about the location of *θ* than the decisions of the closed test procedure of § 2·3. The utility of this additional information will depend strongly on the context. In practice, the primary concern will often be to find lower bounds for the components of *θ* corresponding to the rejected null hypotheses. As this can be achieved using an algorithm that is *O(K*^2^), application to large-scale simultaneous inference problems is, in principle, feasible. However, these lower bounds will only be informative if all hypotheses considered in the second stage of testing are rejected, and even this may be insufficient. In practice, therefore, the lower bounds (12)–(13) are only likely to be useful in relatively small-scale problems. Furthermore, in situations where informative lower confidence bounds are deemed to be more important than the possibility of rejecting as many individual null hypotheses as possible, it would be sensible to use the intervals (14) instead of applying the closed test procedure. For large-scale simultaneous inference problems, an approach based on controlling the false coverage-statement rate (Benjamini & Yekutieli, 2005) may be more appropriate than aiming for a high simultaneous coverage probability.

Extensions to more than two stages and to allow early rejection of hypotheses are straightforward with an appropriate combination function in place of (3). An open question is how best to choose *φ*_∅_(θ*) and $p_{\emptyset }^{\left(2\right)}\left(\theta^{*}\right)$. The tests we use in region (8) are a natural choice but may not be the most powerful.

## Appendix

*Proof of Proposition* 1. With the assumptions in § 3·1, all tests of the form (7) satisfy condition (4), and therefore *C* is a 100(1 − *α*)% confidence set for *θ*. By the monotonicity conditions imposed on the *p*-values, we have $p_{I\cap T_{j}}^{\left(j\right)}\left(\theta^{*}\right)⩽p_{I\cap T_{j}}^{\left(j\right)}$ for all *θ** ∈ Θ_*I*_ (*j* = 1,2; *I* ≠ ∅; *I* ⊆ *T*_1_), so that Θ*_I_* ∩ *C* = ∅ if and only if $Q(p_{I}^{\left(1\right)},p_{I\cap T_{2}}^{\left(2\right)})⩽\alpha$. Therefore, *ψ* =1 if and only if min_*I*⊆*T*_1_,*k*∈*I*_ $Q(p_{I}^{\left(1\right)},p_{I\cap T_{2}}^{\left(2\right)})⩽\alpha$ if and only if ⋃_*I*⊆*T*_1_,*k*∈*I*_ Θ*I* ∩ *C* = ∅. Since ⋃_*I*⊆*T*_1_,*k*∈*I*_ Θ_*I*_ = *H_k_*, we have compatibility.

*Proof of Proposition* 2. First, note the key property that $p_{k,I\cap T_{j}}^{\left(j\right)}\left(\vartheta \right)⩾p_{I\cap T_{j}}^{\left(j\right)}\left(\theta^{*}\right)$ for all *θ** ∈ Θ_*I*_ with $\theta_{k}^{*}⩽\vartheta \left(I\subseteq T_{1};k\in T_{1};j=1,2\right)$.

To show that *C*_1_⊆×_*k*∈*T*_1__ (*l_k_*, ∞), consider any *θ** ∈ Θ \ ×_*k*∈*T*_1__ (*l_k_*, ∞). We must have *θ** ⊆ Θ_*I*_ for some *I* ⊆ *T*_1_ and $\theta_{k}^{*}⩽l_{k}$ for some *k* ∈ *T*_1_. If *k* ∈ *I*, then $\theta_{k}^{*}⩽\min\left(\delta_{k},l_{k,I}\right)$, and (12) implies that $\alpha ⩾Q\{p_{k,I}^{\left(1\right)}\left(\theta_{k}^{*}\right),p_{k,I\cap T_{2}}^{\left(2\right)}\left(\theta_{k}^{*}\right)\}⩾Q\{p_{I}^{\left(1\right)}\left(\theta^{*}\right),p_{I\cap T_{2}}^{\left(2\right)}\left(\theta^{*}\right)\}$. The same inequality follows from $l_{k,I}⩾\theta_{k}^{*}>\delta_{k}$ and (13) if *k* ∉ *I*. Therefore, *θ** ∉ *C*_1_ and *C*_1_ ⊆ ×_*k*∈*T*_1__ (*l_k_*, ∞).

To show that no smaller interval (*l_k_* + *ϵ*, ∞) is possible for any *ϵ* > 0, we must find some *θ** ∈ *C*_1_ with $\theta_{k}^{*}\in \left(l_{k},l_{k}+\epsilon \right)$. Consider a subset *I* ⊆ *T*_1_ such that *l_k_* = *l_k,I_* and therefore $Q\{p_{k,I}^{\left(1\right)}\left(\vartheta \right),p_{k,I\cap T_{2}}^{\left(2\right)}\left(\vartheta \right)\}>\alpha$ for all ϑ > *l_k_*. If *k* ∈ *I* or, equivalently, *l_k_* < *δ_k_*, take any $\theta_{k}^{*}\in \left(l_{k},\min\{\delta_{k},l_{k}+\epsilon \}\right)$. If *k* ∉ *I* or, equivalently, *l_k_* ⩾ *δ_k_*, take any $\theta_{k}^{*}\in \left(l_{k},l_{k}+\epsilon \right)$. Now consider a parameter vector $\xi^{I,k}=(\xi_{1}^{I,k},\ldots ,\xi_{K}^{I,k})$, where $\xi_{k}^{I,k}=\theta_{k}^{*}$, $\xi_{i}^{I,k}=\delta_{i}$ for *k* ≠ *i* ∈ *I*, and $\xi_{i}^{I,k}>\delta_{i}$ for *i* ∉ *I* ∪ {*k*}. All such parameter vectors *ξ*^*I,k*^ are contained in Θ_*I*_, and

$$\alpha <Q\left\{p_{k,I}^{\left(1\right)}\left(\theta_{k}^{*}\right),p_{k,I\cap T_{2}}^{\left(2\right)}\left(\theta_{k}^{*}\right)\right\}=\lim_{\xi_{i}^{I,k}\to \infty ,i\notin I\cup \{k\}}Q\left\{p_{I}^{\left(1\right)}\left(\xi^{I,k}\right),p_{I\cap T_{2}}^{\left(2\right)}\left(\xi^{I,k}\right)\right\}.$$

Thus there exists some such *ξ*^*I,k*^ ∈ *C*_1_ and hence *C*_1_ is not contained in this smaller product of intervals.

Finally, *H_k_* ∩×_*k*∈*T*_1__ (*l_k_*, ∞) = ∅ if and only if *l_k,I_* ⩾ *δ_k_* for *I* ⊆ *T*_1_. if and only if $Q\{p_{k,I}^{\left(1\right)}\left(\delta_{k}\right)$, $p_{k,I\cap T_{2}}^{\left(2\right)}\left(\delta_{k}\right)\}=Q\{p_{I}^{\left(1\right)},p_{I\cap T_{2}}^{\left(2\right)}\}⩽\alpha$ for *I* ⊆ *T*_1_ and *k* ∈ *I*, if and only if *ψ_k_* = 1.
